# Pneumolysin contributes to dysfunction of nasal epithelial barrier for promotion of pneumococcal dissemination into brain tissue

**DOI:** 10.1128/msphere.00655-24

**Published:** 2024-09-30

**Authors:** Yuki Takahara, Tomoko Sumitomo, Masamitsu Kono, Moe Takemura, Yukako Akamatsu, Yujiro Hirose, Masaya Yamaguchi, Masanobu Nakata, Muneki Hotomi, Shigetada Kawabata

**Affiliations:** 1 Department of Microbiology, Osaka University Graduate School of Dentistry, Osaka, Japan; 2 Department of Fixed Prosthodontics and Orofacial Function, Osaka University Graduate School of Dentistry, Osaka, Japan; 3 Department of Oral Microbiology, Graduate School of Biomedical Sciences, Tokushima University, Tokushima, Japan; 4 Department of Otorhinolaryngology—Head and Neck Surgery, Wakayama Medical University, Wakayama, Japan; 5 Department of Oral Surgery, Rinku General Medical Center, Izumisano, Osaka, Japan; 6 Division of Special Care Dentistry, Osaka University Dental Hospital, Osaka, Japan; 7 Bioinformatics Research Unit, Osaka University Graduate School of Dentistry, Osaka, Japan; 8 Bioinformatics Center, Research Institute for Microbial Diseases, Osaka University, Osaka, Japan; 9 Center for Infectious Diseases Education and Research, Osaka University, Osaka, Japan; 10 Department of Oral Microbiology, Kagoshima University Graduate School of Medical and Dental Sciences, Kagoshima, Japan; University of Kentucky College of Medicine, Lexington, Kentucky, USA

**Keywords:** *Streptococcus pneumoniae*, pneumolysin, bacterial meningitis, non-hematogenous route

## Abstract

*Streptococcus pneumoniae* is one of the major pathogens responsible for bacterial meningitis and neurological sequelae. The present study was conducted to identify a non-hematogenous route used by *S. pneumoniae* to gain access to brain tissue without causing bacteremia or pneumonia, as well as bacterial and host factors involved in this process. To investigate the molecular mechanisms and dissemination pathways of pneumococcal infection in brain tissue, mice were intranasally inoculated with *S. pneumoniae* strain EF3030, a clinical isolate from a patient with otitis media. Pneumococci were isolated from the frontal olfactory bulb, caudal cerebrum, and cerebellum, with neither bacteremia nor pneumonia observed in the present model. Immunostaining imaging revealed the presence of *S. pneumoniae* organisms in olfactory nerve fibers. Knockout of the *ply* gene encoding pneumolysin (PLY) markedly compromised the ability of the bacterial organisms to disseminate into brain tissue, whereas the dissemination efficiency of the complemented strain was restored to nearly the same level as the wild type. Notably, distinct upregulation of Gli1 and Snail1, which are involved in the transcriptional repression of junctional proteins, along with downregulation of E-cadherin, was detected in nasal lavage samples from mice infected with the wild-type or complemented strain, but not in those from mice infected with the *ply* mutant. Taken together, the present findings indicate that PLY induces Gli1-Snail1-dependent dysfunction of the nasal epithelial barrier, thus allowing pneumococcal dissemination to brain tissue that occurs in a non-hematogenous manner.

**IMPORTANCE:**

Bacterial meningitis, considered to be caused by bacteremia, can lead to blood–brain barrier disruption and bacterial dissemination into the central nervous system. Despite the availability of intravenously administered antibiotics with cerebrospinal fluid transferability, bacterial meningitis remains associated with high rates of morbidity and mortality. Here, we utilized *Streptococcus pneumoniae* strain EF3030, clinically isolated from otitis media, for the construction of a murine infection model to investigate the molecular mechanisms by which nasally colonized pneumococci disseminate into brain tissue. The obtained findings indicate that pneumolysin (PLY) induces Gli1-Snail1-dependent dysfunction of the nasal epithelial barrier, which facilitates pneumococcal dissemination to brain tissue in a non-hematogenous manner. Our results support the existence of an alternative route by which *S. pneumoniae* can reach the central nervous system and indicate the need for the development of novel therapeutic strategies, which would be an important contribution to the clinical management of bacterial meningitis.

## INTRODUCTION

Bacterial meningitis is a worldwide medical concern and causes significant morbidity and mortality. The three most common pathogens, *Haemophilus influenzae*, *Neisseria meningitidis*, and *Streptococcus pneumoniae*, account for more than 80% of affected cases (1). In particular, *S. pneumoniae* is the predominant etiological agent for bacterial meningitis across all regions and age groups, with the highest incidence rates noted in young children and the elderly (2–4). Although *S. pneumoniae* is a common human pathogen that colonizes the nasopharynx and upper respiratory tract of healthy individuals, it can cause a variety of diseases, including otitis media, pneumonia, septicemia, and bacterial meningitis. According to the World Health Organization, this bacterium is the fourth most frequent microbial cause of fatal infections; thus, it is important to understand its underlying pathogenicity (5, 6).

It has long been considered that pneumococcal meningitis is attributed to bacteria that initially entered the lower respiratory tract and eventually caused pneumonia and septicemia. Septicemia ultimately results in disruption of the blood–brain barrier, allowing the bacteria to disseminate into the central nervous system (CNS) (7). Despite the availability of intravenously administered antibiotics with cerebrospinal fluid transferability, pneumococcal meningitis remains associated with high rates of morbidity and mortality. Indeed, approximately 25% of cases of neonatal bacterial meningitis failed to detect the causative agent in blood cultures (7–9). Another study found that a *galU*-deficient mutant of *S. pneumoniae*, which cannot survive in the blood when given intranasally, can still cause meningitis in mice (10). These observations suggest that *S. pneumoniae* can gain access to the CNS without causing pneumonia and peripheral blood infection. A previous study showed that the bacterium has an ability to bind to gangliosides and invade brain tissue via retrograde axonal transport (11). Moreover, free sialic acid enhances pneumococcal invasion into the olfactory bulb without causing blood infection, highlighting the significant role of sialic acid availability in pneumococcal pathogenesis (12). Recently, another group also reported that pneumococci colonized in the nasopharynx rapidly translocate across the cribriform plate to invade the outer meninges (13). Although these results support the hypothesis that *S. pneumoniae* directly disseminates from the nasopharynx to brain tissue, the underlying molecular mechanisms, together with factors related to dysfunction of the nasal epithelial barrier, remain largely unknown.

The olfactory epithelium, located inside the nasal cavity, serves as the first line of defense against a variety of pathogens. The adherens junctions and tight junctions are both essential for maintaining cell-to-cell and cell-to-matrix connections (14, 15), and their disruption can potentially allow invasion of bacterial cells. Pathogens have evolved and developed specific strategies to target intercellular junctions and their components. In severe invasive pneumococcal infections, *S. pneumoniae* invades underlying sterile tissue by translocating across the epithelial barrier while evading host defense mechanisms. Cholesterol-dependent cytolysins (CDCs) constitute a large family of pore-forming toxins that exclusively target cholesterol-containing membranes. CDC-mediated pore formation activates cytoskeletal rearrangement and calcium influx, resulting in dysfunction of cellular junctions. Pneumolysin (PLY), a representative CDC released from *S. pneumoniae* through autolysis, has been shown to play crucial roles in the pathogenesis of pneumococcal meningitis (16). Another study found that PLY hijacks the endocytic machinery to enhance neuroinflammatory response in pneumococcal meningitis cases (17). As a prerequisite for causing pneumococcal meningitis, *S. pneumoniae* must overcome the host epithelial barrier, which leads to systemic dissemination from the nasopharynx with evasion of host defense mechanisms. A previous report noted that *S. pneumoniae* was found to induce cleavage and mislocalization of the junctional protein E-cadherin in a PLY-dependent manner (18), though the precise mechanism has yet to be completely elucidated. *S. pneumoniae* and *H. influenzae* infections have been reported to induce activation of both the p38 mitogen-activated protein kinase (MAPK) and TGF-β signaling cascades, leading to the upregulation of Snail1 and the downregulation of claudins, components of tight junctions (19). Additionally, PLY has been demonstrated to induce innate immune responses in the nasopharynx by activating MAPK via TLR4 (20).

For the present study, a murine model of non-hematogenous pneumococcal meningitis was used to investigate the molecular mechanisms by which nasally colonized *S. pneumoniae* disseminates into brain tissue. The obtained findings indicate that PLY induces upregulation of Gli1–Snail1, the transcriptional repressors of junctional proteins, leading to dysfunction of the nasal epithelial barrier, which allows pneumococcal dissemination from the nasopharynx to brain tissue via a non-hematogenous route.

## RESULTS

### *S. pneumoniae* disseminates into brain tissues without causing severe pneumonia and bacteremia

**Fig 1:**
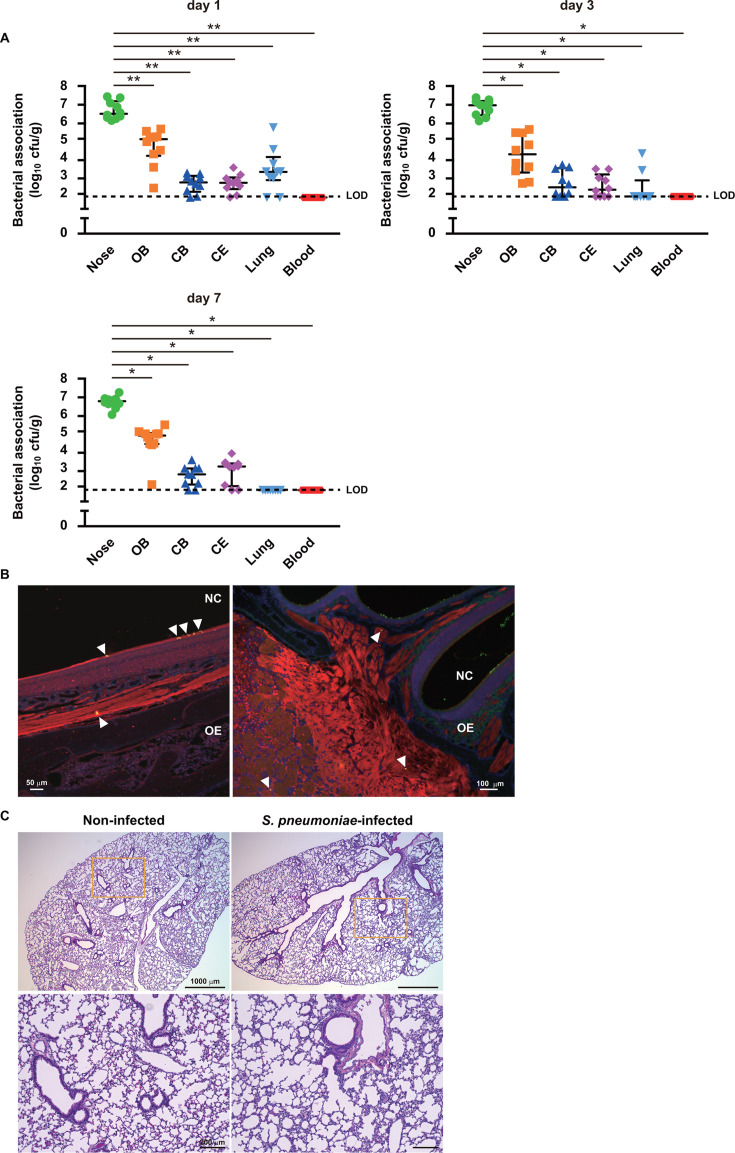
*S. pneumoniae* colonized in nasopharynx disseminates into brain tissue through a non-hematogenous route. Mice were intranasally infected with *S. pneumoniae* EF3030 (WT, 1 × 10^7^ CFU in 10 μL of phosphate-buffered saline [PBS]). (A) Bacterial burden in the nasopharynx (nose), olfactory bulb (OB), cerebrum (CB), cerebellum (CE), lung tissues, and blood was assessed at days 1, 3, and 7 following infection. Dots indicate individual mice. Horizontal lines show median values and interquartile range. Dotted line shows the limit of detection (LOD). Data shown are representative of at least three separate experiments, with 10 mice used per group. Statistically significant differences were evaluated using one-way analysis of variance (ANOVA), followed by Tukey’s multiple-comparison test. *P < 0.0001, **P < 0.001. (B) Brain section obtained from mice nasally infected with EF3030 were subjected to immunofluorescence staining at day 3 following infection. Pneumococci were labeled with an anti-serotype 19 capsule antibody, followed by incubation with an Alexa Fluor 488-conjugated antibody. The olfactory nerve was labeled with an anti-olfactory marker protein and Alexa Fluor 594-conjugated antibodies, and cell nuclei were stained with 4′,6-diamidino-2-phenylindole (DAPI). Obtained tissue sections were analyzed using confocal laser microscopy. Arrowheads indicate pneumococcal association with olfactory neurons. Nasal cavity (NC) and olfactory epithelium (OE). Data shown are representative of at least three separate experiments. (C) Lung tissues obtained from non-infected and infected mice at day 3 following infection were subjected to hematoxylin and eosin (HE) staining. Magnified images of boxed areas in upper panels are shown in lower panels. Data shown are representative of at least three separate experiments, with three mice used per group.

*S. pneumoniae* has been shown to invade the CNS through a non-hematogenous route (11–13). To examine whether *S. pneumoniae* colonized in the nasopharynx disseminate into brain tissue without causing pneumonia and/or bacteremia, bacterial dissemination as well as lung pathogenesis were evaluated using a murine model of pneumococcal intranasal colonization. The numbers of bacteria colonized in the nasopharynx were nearly identical at 1, 3, and 7 days after infection, though significantly more were isolated from the frontal olfactory bulb (OB) than from the caudal cerebrum (CB) and cerebellum (CE) (Fig. 1A). Immunostaining visualized *S. pneumoniae* colocalized with olfactory neurons within the epithelium and subsequently disseminating along the neurons, entering brain tissues at the cribriform plate (Fig. 1B; Fig. S1). No detectable bacteria were recovered from blood at time points tested (Fig. 1A). Furthermore, viable pneumococci were isolated from lung tissues at 1 and 3 days after infection, while they were completely eliminated by day 7 (Fig. 1A). Histopathological analysis also revealed no apparent inflammation, such as perivascular or peribronchial lymphocytic cuffing, nor injury in both non-infected and infected lung tissues (Fig. 1C). These findings support the notion that *S. pneumoniae* can invade the CNS via the olfactory nerve without causing severe pneumonia or bacteremia, using a non-hematogenous route.

### PLY contributes to olfactory epithelial barrier dysfunction

**Fig 2:**
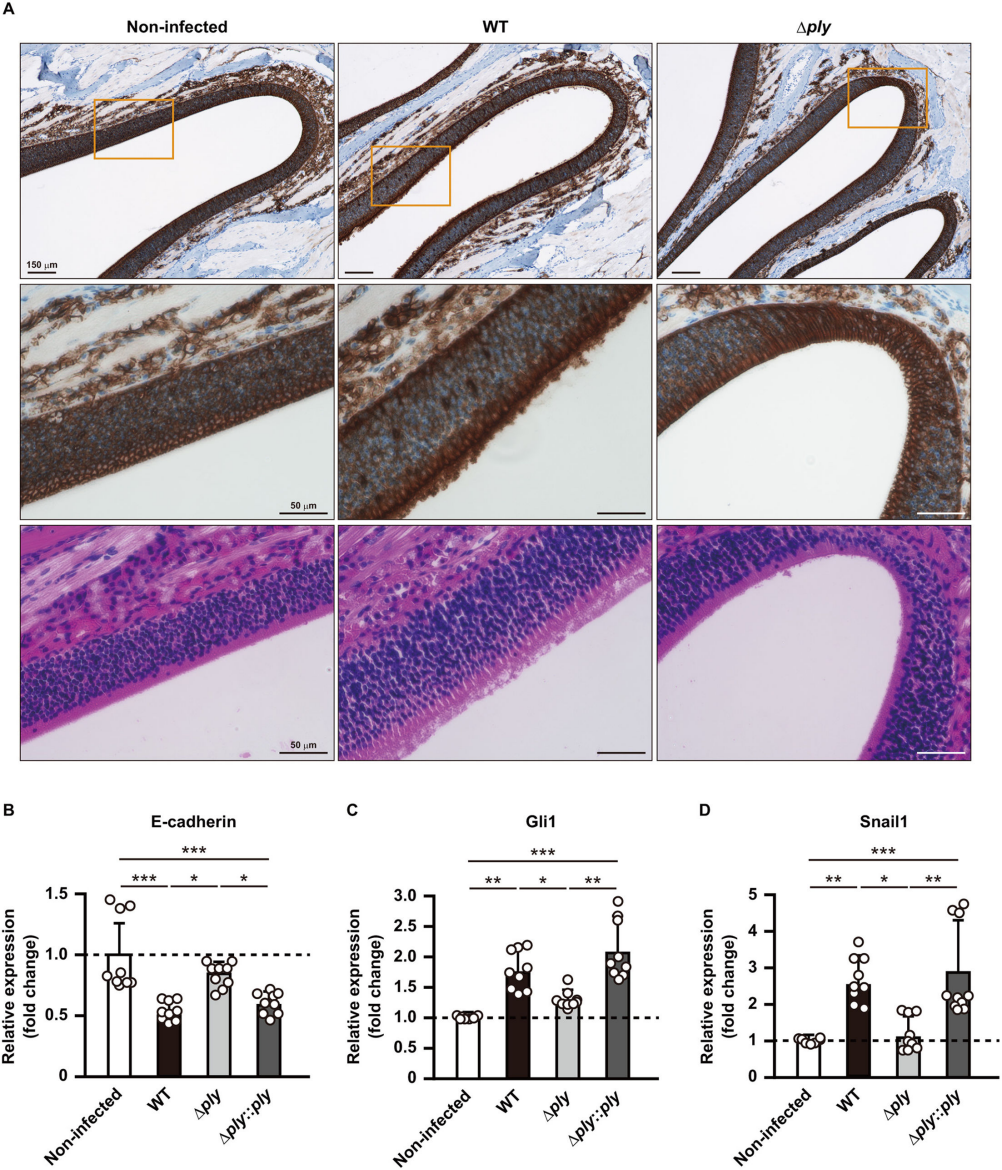
PLY induces Gli1- and Snail1-dependent dysfunction of the nasal epithelial barrier. (A) Brain tissues were obtained from mice infected with *S. pneumoniae* EF3030 (WT, 1 × 10^7^ CFU in 10 μL of PBS), or an isogenic *ply* knockout strain (Δ*ply*), then subjected to immunohistochemistry using an anti-E-cadherin antibody and HE staining performed on day 3 following infection. Magnified images of boxed areas in the upper panels are presented in the middle panels. Data shown are representative of at least three separate experiments, with five mice per group. (B–D) Transcriptional levels of genes encoding (**B**) E-cadherin, (**C**) Gli1, and (**D**) Snail1 in nasal lavage fluid were determined at 3 days following infection using real-time RT-PCR. A *gapdh* transcript served as the internal control. Dots indicate individual mice. Values for expression ratios pooled from three independent examinations are presented as the mean ± SD. Transcriptional levels are presented as relative expression normalized to that seen in non-infected tissues. *P < 0.05, **P < 0.01, ***P < 0.001.

For establishment of bacterial meningitis via a non-hematogenous route, *S. pneumoniae* must initially colonize the nasopharynx, then transmigrate to the olfactory epithelial barrier, and evade the host immune system. Moreover, when asymptomatically colonized in the nasopharynx, *S. pneumoniae* has been shown to induce necroptosis in nasopharyngeal epithelial cells through PLY activity (21). To explore the potential contribution of PLY to the dysfunction of the olfactory epithelial barrier induced by *S. pneumoniae*, E-cadherin distribution in the olfactory epithelium was assessed at 3 days after bacterial administration (Fig. 2A, upper and middle panels). In non-infected mice, E-cadherin immunoreactivity was continuously detected along neighboring epithelial cell borders and between adjacent olfactory cells. In contrast, redistribution of E-cadherin to both the cytosol and junctions was observed in the olfactory epithelium of mice infected with the wild type (WT) strain. On the other hand, typical E-cadherin expression was detected in olfactory epithelium of mice infected with the *ply* mutant strain, similar to that observed in non-infected mice. Moreover, histopathological evaluation findings obtained using hematoxylin and eosin (HE) staining revealed various levels of damage in the olfactory epithelium of mice following WT infection, including loss of surface microvilli and substantial structural disorganization, which were not observed in non-infected or *ply* mutant strain-infected mice (Fig. 2A, lower panels). To clarify the correlation between the dysfunction of the expression level of E-cadherin, the nasal epithelium was assessed 3 days after bacterial administration (Fig. 2B). Quantitative RT-PCR analysis revealed a significant decrease in E-cadherin expression in the nasal lavage fluid of mice infected with the WT strain. Interestingly, that decreased expression was substantially abated by knockout of the *ply* gene and nearly restored by complementation. Snail1, a global repressor of genes encoding junctional proteins, is positively regulated by Gli1 through activation of the MAPK pathway (22). The present results showed distinct upregulation of Gli1 and Snail1 in nasal lavage samples obtained from mice infected with the WT or complemented strain, while those from mice infected with the *ply* mutant strain were nearly identical to those from non-infected mice (Fig. 2C and D), indicating regulation by *S. pneumoniae* in a PLY-dependent manner. Additionally, it is suggested that the Gli1–Snail1 axis contributes to PLY-mediated dysfunction of the nasal and olfactory epithelial barriers.

### PLY is a critical factor for pneumococcal dissemination into brain tissues

**Fig 3:**
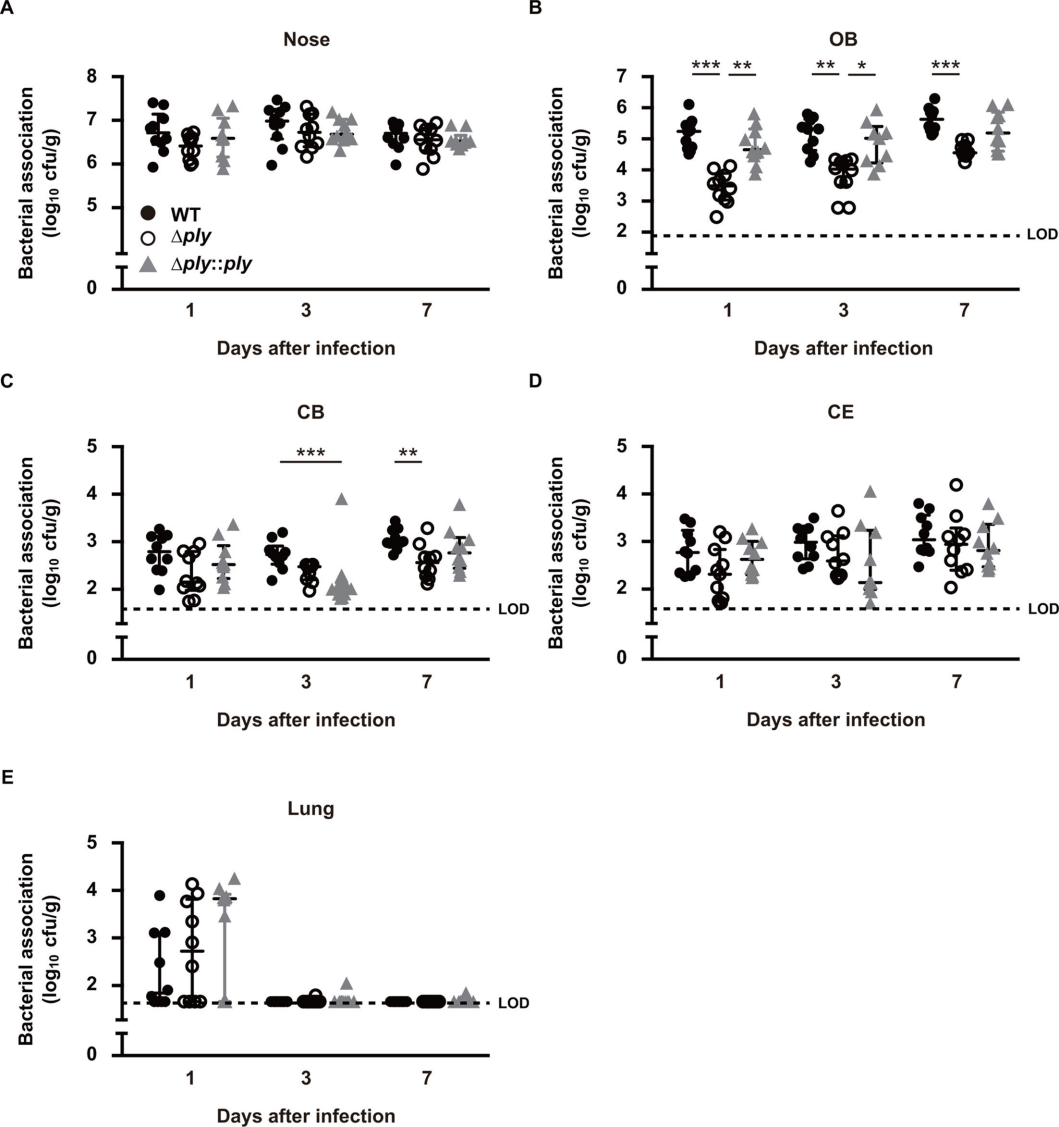
PLY contributes to pneumococcal dissemination into the brain tissue. Mice were intranasally infected with *S. pneumoniae* strains (1 × 10^7^ CFU in 10 μL of PBS), then bacterial burden in the (**A**) nasopharynx (nose), (**B**) OB, (**C**) CB, (**D**) CE, and (**E**) lung tissues was assessed at 3 days following infection. Dots indicate individual mice. Horizontal lines show median values and interquartile range. Dotted line shows the limit of detection (LOD). Data shown are representative of at least three separate experiments, with 10 mice used per group. Statistically significant differences were evaluated using one-way ANOVA, followed by Tukey’s multiple-comparison test. *P < 0.05, **P < 0.01, ***P < 0.001.

Subsequently, we investigated the correlation of PLY-mediated dysfunction of the olfactory epithelial barrier with pneumococcal dissemination from the nasopharynx to brain tissues. Bacterial colonization in the nasal cavity was nearly identical among the tested *S. pneumoniae* strains (Fig. 3A). However, mice infected with the *ply* knockout mutant exhibited significantly reduced bacterial colonization in the OB at days 1, 3, and 7 following infection compared to those infected with the WT or complemented strain (Fig. 3B). While the rates of pneumococcal dissemination to the CB were found to be diminished by *ply* knockout at 7 days following infection, bacterial numbers recovered from the CE were equivalent among the tested strains (Fig. 3C and D). *S. pneumoniae* colonizing the nasopharynx has been shown to be disseminated via both olfactory and trigeminal nerve pathways (11). This suggests that most pneumococci recovered from the CB and CE likely undergo translocation through the trigeminal nerve in a PLY-independent manner. Furthermore, bacterial burden was observed in the lungs of mice infected with the tested strains, though *S. pneumonia*e was found to be nearly eliminated by day 3 (Fig. 3E).

**Fig 4:**
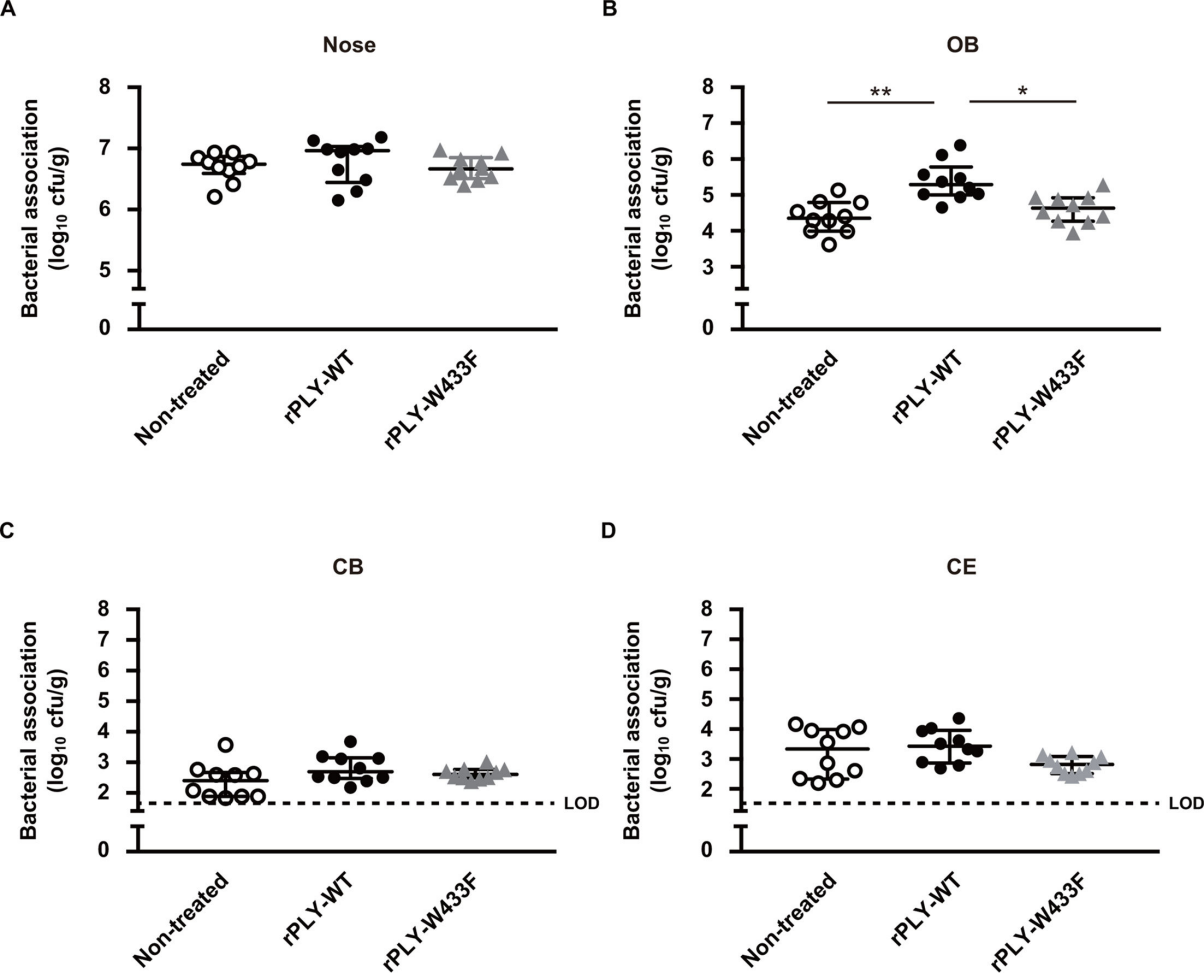
Pore-forming activity of PLY critical for pneumococcal dissemination into the brain tissue. Mice were intranasally administered with the vehicle, recombinant PLY (rPLY-WT), or a non-pore-forming toxoid with the W433F point mutation (rPLY-W433F) 24 h before infection with the *ply* mutant. On day 3 following infection, bacterial colonization in the (**A**) nasopharynx (nose), (**B**) OB, (**C**) CB, and (**D**) CE tissues was assessed. Dots indicate individual mice. Horizontal lines show median values and interquartile range. Dotted line shows the limit of detection (LOD). Data shown are representative of at least three separate experiments, with 10 mice used per group. Statistically significant differences were evaluated using one-way ANOVA, followed by Tukey’s multiple-comparison test. *P < 0.05, **P < 0.001.

To further clarify the role of PLY in pneumococcal dissemination into the CNS through a non-hematogenous route, mice were intranasally administered recombinant PLY (rPLY-WT), the non-pore-forming toxoid with the W433F point mutation (rPLY-W433F), or the vehicle (non-treated), at 24 h before infection with the *ply* mutant. Despite nearly identical pneumococcal colonization in the nasal cavity under each condition, nasal administration of rPLY-WT substantially increased bacterial burden by the *ply* mutant in the OB segment, whereas no significant difference was observed in mice treated with rPLY-W433F (Fig. 4A and B). Additionally, there was no significant difference in pneumococcal dissemination to the CB and CE segments between rPLY-WT and rPLY-W433F administrations (Fig. 4C and D). These findings suggest that the pore-forming activity of PLY is crucial for olfactory epithelial barrier dysfunction and pneumococcal invasion into the OB segment.

### PLY-dependent pneumococcal dissemination leads to increased chemokine expression in brain tissues

**Fig 5:**
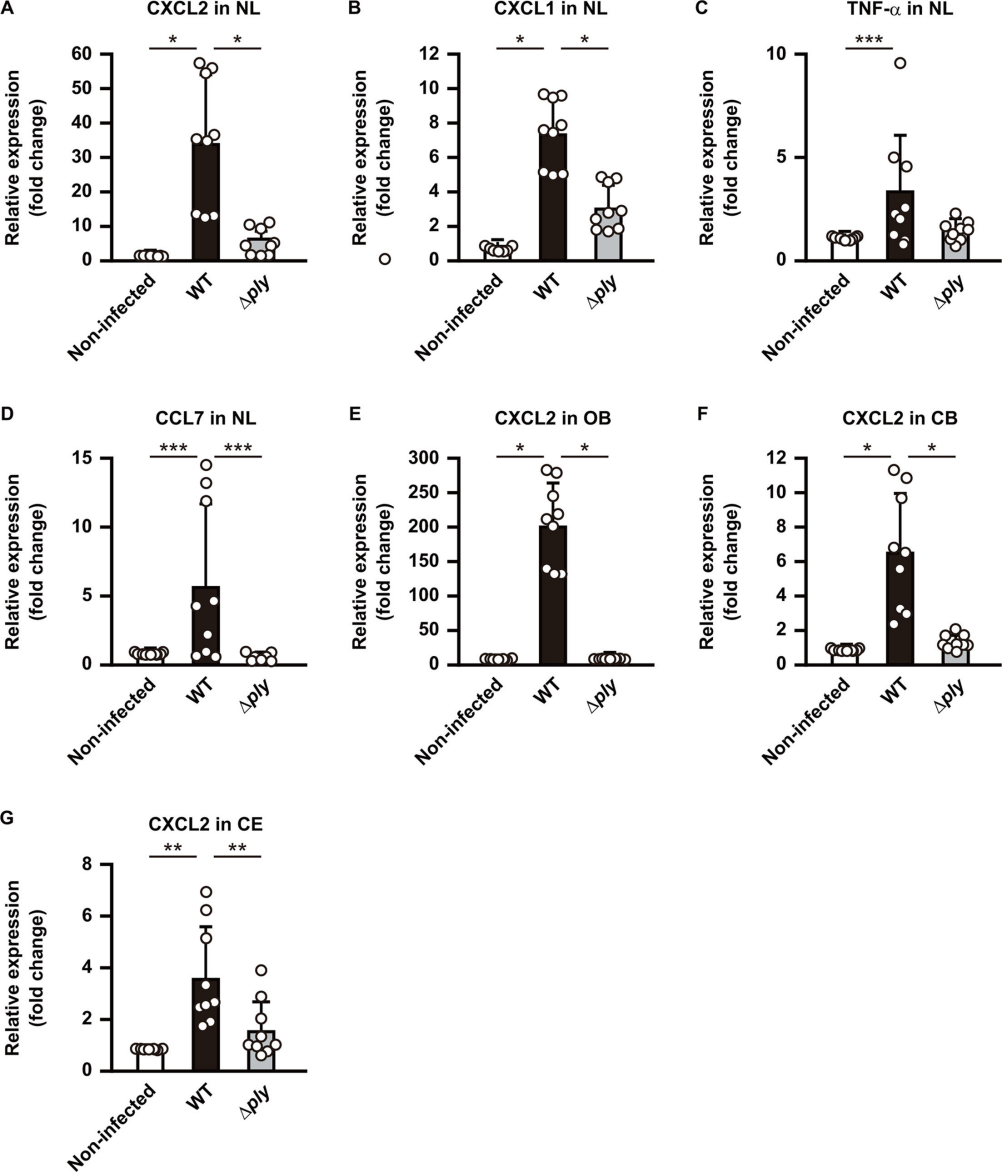
PLY-dependent inflammatory response in brain tissues. (A–D) Real-time RT-PCR assays were performed on day 3 following infection to analyze transcriptional levels of genes encoding (**A**) CXCL2, (**B**) CXCL1, (**C**) TNF-α, and (**D**) CCL7 in the nasal lavage fluid (NL) of mice infected with *S. pneumoniae* strains. (E–G), Transcriptional levels of the CXCL2 gene in (**E**) OB, (**F**) CB, and (**G**) CE were assessed at 3 days following infection. A *gapdh* transcript served as the internal control. Dots indicate individual mice. Values for expression ratios pooled from three independent examinations are presented as the mean ± SD. Transcriptional levels are presented as relative expression normalized to that of non-infected tissues. *P < 0.0001, **P < 0.001, ***P < 0.05.

Inflammatory response in the cerebrospinal fluid (CSF) is one of the indicators of pneumococcal meningitis severity (23). To investigate the role of PLY in proinflammatory response, induction of cytokine/chemokine production was assessed in nasal lavage fluid and each brain segment obtained from mice infected with the WT or *ply* mutant. At 3 days after the initial infection, significant increases in *Tnfa*, *Cxcl1*, *Cxcl2*, and *Ccl7* mRNA levels were noted in the nasal lavage fluid obtained from mice infected with the WT, compared to non-infected mice (Fig. 5A through D). On the other hand, infection with the *ply* mutant did not increase the mRNA levels, compared to infection with the WT. Additionally, in mice infected with the WT, the mRNA level of *Cxcl2* was significantly increased in the OB, CB, and CE segments compared to non-infected mice (Fig. 5E through G), while no change in levels of *Tnfa*, *Cxcl1*, and *Ccl7* expression were observed (data not shown). The *Cxcl2* expression in mice infected with the *ply* mutant was at the same level as that seen in non-infected mice, indicating a strong correlation between PLY-mediated bacterial dissemination to brain tissues and induction of a proinflammatory response.

## DISCUSSION

Bacterial meningitis caused by *S. pneumoniae* is generally considered to occur when pneumococci give rise to focal pneumonia in the lower respiratory tract, which is preceded by bacteremia and subsequent disruption of the blood–brain barrier. As recently reported, *S. pneumoniae* and other bacteria responsible for bacterial meningitis, including *Neisseria meningitidis* and *Listeria monocytogenes*, utilize a nose-to-brain pathway for successful invasion into the CNS through a non-hematogenous route (11–13, 24, 25). To gain insight into the mechanism by which *S. pneumoniae* disseminates from the nasopharynx to the CNS, the present study was conducted to identify bacterial and host factors involved in that process. The findings show that PLY induces Snail1-dependent dysfunction of the nasal epithelial barrier, thus allowing pneumococcal dissemination to the brain tissue in a non-hematogenous manner.

PLY is a key virulence factor of *S. pneumoniae* and produced by virtually all clinical isolates. As for the pathogenesis of pneumococcal meningitis, PLY plays a crucial role by facilitating bacterial penetration of the blood–brain barrier and contributing to loss of tight junctions (26, 27). Results obtained in the present study indicated upregulation of the *GLI1* and *SNAI1* genes, along with downregulation of the E-cadherin gene in the nasal lavage fluid obtained from mice infected with the WT strain (Fig. 2B through D). Interestingly, the expression profiles of those genes in mice infected with the *ply* mutant closely resembled those in non-infected mice (Fig. 2B through D). The olfactory epithelium is composed of three cell types, olfactory, supporting, and basal cells, which are interconnected by cell-to-cell adherence proteins such as E-cadherin. Sonic Hedgehog (Shh) functions as a morphogen, providing positional information to control cell fate decisions and organize the pattern of differentiation (28). Three ligands, including Shh, Ihh, and Dhh, bind to the transmembrane receptor Patched1 (PTCH1), resulting in the release of Smoothened (SMO), which mediates the downstream activation of Gli1 (29). As a result, Shh-mediated activation of Gli1 induces Snail1; represses junctional proteins; including E-cadherin, ZO-1, and claudins; and enhances the nuclear translocation of β-catenin, which induces epithelial–mesenchymal transition (30). The present findings provide additional evidence showing that PLY-mediated Gli1–Snail1 axis is crucial for dysfunction of the olfactory epithelial barrier, which subsequently leads to pneumococcal dissemination to the CNS through a non-hematogenous route. A previous study showed that Gli1 cross-activated by the MAPK and PI3K pathways in *Salmonella enterica* serovar Typhimurium-infected epithelial cells induces Snail expression, leading to disruption of the epithelial barrier (31). Furthermore, the cytolytic activity and diverse immunomodulatory properties of PLY have been demonstrated to influence the balance between pathogen and host, through various signal transduction pathways, including the MAPK pathway (32); thus, an unidentified pathway activated by PLY may induce the Gli1–Snail1 axis, leading to dysfunction of the mucosal epithelial barrier.

The PLY pore formation induces consecutive changes in cellular homeostasis, which is triggered by a robust influx of cytosolic calcium (Ca^2+^) (33). Host cell responses vary depending on the PLY concentration, the extent of plasma membrane damage and intracellular Ca^2+^ overload changes, and then leads to activation of either cell death or survival, and damage or repair of related pathways (32). In cases of brain infection with *S. pneumoniae*, PLY induces neuronal cell death and targets astrocytes, causing rearrangement of the cytoskeleton and alterations in the cell shape of astrocytes (34). PLY-dependent astrocyte cell death has been demonstrated to be mediated by connexin 43, a gap junction protein that forms hemichannels (35). This amplifies ATP release and cytosolic Ca^2+^ influx, ultimately resulting in astrocyte depletion and destabilization of the blood–brain barrier. In airway epithelial cells, Ca^2+^ influx mediated by TLR2 signaling activates calpains, known to be Ca^2+^-dependent host cysteine proteases (36). These calpains then target junctional proteins, such as occludin and E-cadherin. Indeed, findings obtained in our previous studies demonstrated that *Streptococcus pyogenes* and influenza A virus infections induce Ca^2+^-dependent calpain activation, resulting in destabilization of paracellular junctions and facilitation of subsequent bacterial invasion into deeper tissues (37, 38). Therefore, in addition to the Gli1–Snail1 axis, specific proteolytic cleavage events mediated by PLY, such as those facilitated by calpain, may result in targeting and inactivation of E-cadherin in *S. pneumoniae*-colonized olfactory epithelial cells.

There are at least five pathways as a non-hematogenous route of colonization from the nasal mucosal epithelium to the brain through the foramina of the cribriform plate. Those pathways include the axon of a nerve-mediated pathway (anterograde along olfactory axons or retrograde along trigeminal axons), an olfactory ensheathing cell-mediated route, perineuronal spaces of nerve transport, a lymphatic delivery pathway, and intraperiosteal and intrathecal transport pathways. Although sialic acid, an extracellular glycopeptide of olfactory nerve sheath cells, has been shown to promote non-hematogenous pneumococcal delivery to the olfactory bulb, retrograde dissemination is likely challenging due to drainage of tissue fluids, such as lymph or CSF, from the cranium into the subarachnoid space (12, 39). On the other hand, it is possible that bacteria could invade by using gaps between connective tissue that binds nerve fibers and the nerve extramembrane. The present results indicating co-localization of *S. pneumoniae* with the olfactory maker protein, a marker of the olfactory nerve system, support the notion that *S. pneumoniae* utilizes bundles of axons in the lamina propria, the olfactory bulb, and the olfactory receptor cells in the olfactory epithelium for bacterial invasion into the CNS.

A wide variety of cytokines and chemokines have been detected in the CSF of pneumococcal meningitis patients (23, 40). TNF-α, CXCL1, and CXCL2 are important early-inflammatory cytokines/chemokines that attract leukocytes to the subarachnoid space (7). The leukocyte recruitment, particularly neutrophils, into the CSF and brain is a key pneumococcal meningitis indicator. Significantly increased TNF-α, CXCL1, and CXCL2 mRNA levels were detected in the nasal lavage fluid obtained from infected mice (Fig. 5A through C). However, despite recovery of numerous pneumococci from each brain segment, including OB, CE, and CB (Fig. 1), no apparent increase in TNF-α, CXCL1, or CXCL2 expression was observed in brain tissues of infected mice (data not shown). Similar findings have been reported regarding the expression of CCL7, a PLY-dependent monocyte and neutrophil chemoattractant (41, 42). Results obtained with the present mouse model might have been due to the intact blood–brain barrier. Moreover, it was recently shown that PLY induces the activation of nociceptors and release of neuropeptide calcitonin gene-related peptide (CGRP) in trigeminal neurons (43). Interaction of CGRP with receptor activity-modifying protein 1 (RAMP1) in the meningeal macrophages suppresses macrophage chemokine expression, neutrophil recruitment, and antimicrobial defenses during hematogenous pneumococcal meningitis. Mannose receptor C-type 1 (MRC1), a meningeal macrophage marker, has been reported to bind to PLY, which downregulates inflammation and enhances bacterial survival at the infection site (44), while *S. pneumoniae* strain EF3030 has been detected in the trigeminal ganglion following nasal infection (11). Therefore, CGRP–RAMP1 signaling might also be associated with repression of proinflammatory responses in the brain tissues. On the other hand, PLY-dependent upregulation of the neutrophil-attracting chemokine CXCL2 in the OB, CB, and CE segments was observed in infected mice (Fig. 5). Although our data imply that the toxic activity of PLY induces the upregulation of CXCL2, bacterial loads in each brain section differed depending on the examined strains. Thus, additional bacterial factors may be involved in the process. Mobilization and recruitment of neutrophils is primarily dependent on CCL2, a monocyte-attracting chemokine that is also found in elevated levels in infected brain tissues.

The present study examined the mechanism by which *S. pneumoniae* uses a non-hematogenous route to access brain tissue without causing bacteremia or pneumonia. However, no apparent symptoms or pathological features of meningitis were observed in a murine model (data not shown). PLY-dependent pathological features of meningitis, including ependymal damage, meningeal inflammation, and neuronal damage, have been identified in animal models established by direct bacterial inoculation into the CNS (45, 46). Additionally, strain D39, a clinical isolate obtained from a patient with pneumonia, has been used as a test strain in several direct injection studies, with the results suggesting that meningeal inflammation and neuroinflammation in animal models are dependent on the strain and administration route. Additional studies are needed to determine the precise role of PLY in the development of pneumococcal meningitis through a non-hematogenous route.

Taken together, the present findings indicate that PLY induces a Snail1-dependent dysfunction of the nasal epithelial barrier, which facilitates pneumococcal dissemination to the brain tissue in a non-hematogenous manner. However, it is important to note that for establishment of pneumococcal meningitis, a non-hematogenous process is not the sole or possibly even the primary route. Clinical reports suggest that nearly all patients with meningitis are bacteremic, indicating that the majority of bacterial meningitis cases may have developed through a hematogenous route. Nevertheless, these results support the existence of an alternative route by which *S. pneumoniae* can reach the CNS and indicate the need for development of novel therapeutic strategies, which would be an important contribution to clinical management for bacterial meningitis.

## MATERIALS AND METHODS

### Bacterial strains and culture conditions

An encapsulated *S. pneumoniae* strain (EF3030, serotype 19F) clinically isolated from a patient with otitis media and its isogenic mutant strains were cultured in Todd–Hewitt broth supplied by Becton, Dickinson and Company (BD) supplemented with 0.2% yeast extract (BD) (THY medium) at 37°C. For selection and maintenance of mutant strains, spectinomycin (FUJIFILM) and erythromycin (Sigma-Aldrich) were added to the medium at 100 and 1 µg/mL, respectively. *Escherichia coli* strain BL21-gold (DE3) (Agilent Technologies) was used as a host for derivatives of the plasmid pGEX-6P-1 (Cytiva). All *E. coli* strains were cultured in Luria–Bertani (LB) medium (Nacalai Tesque) at 37°C with agitation. For selection and maintenance of the *E. coli* mutant strains, ampicillin was added to the medium at 100 µg/mL.

### Preparation of *S. pneumoniae* mutant strains and recombinant proteins

Inactivation of the *ply* gene was performed by transforming strain EF3030 with a linear DNA fragment containing a spectinomycin resistance gene (*aad9*), flanked by the upstream and downstream sequences of the *ply* gene, as previously reported (47).

For construction of a *ply*-complemented strain, a linear DNA fragment containing an erythromycin resistance gene and the *ply* gene was introduced into the noncoding region between the EF3030_02320 and EF3030_02325 genes on the chromosome of the Δ*ply* strain (48). Utilizing genomic DNA of *S. pneumoniae* EF3030 as a template, amplification of the downstream sequence of EF3030_02320, the *ply* gene with its ribosomal binding site and promoter region, and the upstream sequence of EF3030_02325 was performed. The erythromycin resistance gene was also amplified using pMSP3545 plasmid (49). The four resultant PCR-amplified fragments were linked via overlapping PCR, then transformed into a Δ*ply* mutant. Correct insertion of the fragment into genomic DNA was confirmed by site-specific PCR.

Recombinant PLY proteins were hyper-expressed in *E. coli* BL21-gold (DE3) using a pGEX-6P-1 vector. N-terminal GST-tagged proteins were purified using a GST gene fusion system (Cytiva). For construction of a non-pore-forming toxoid with a W433F point mutation (50–52), an overlapping PCR strategy was used. All primers used are listed in Table S1.

### Mouse experiments

*S. pneumoniae* strains were grown to the mid-exponential phase, then washed with and resuspended in phosphate-buffered saline (PBS). Female Balb/c mice aged 6 to 8 weeks (Japan SLC, Inc.) were intranasally inoculated with 1 × 10^7^ CFU of *S. pneumoniae* in 10 µL of PBS, delivered as a 5-µL droplet per nostril. For quantification of pneumococcal dissemination, mice were euthanized at 1, 3, or 7 days after infection, then perfused with 15 mL of PBS via intracardial puncture with a 22-gauge butterfly needle. The nasal mucosa, olfactory bulb, cerebrum, cerebellum, and lung tissues were immediately collected into tubes containing sterile PBS and zirconia/silica beads (diameter 1.0 mm, BioSpec Products) and homogenized using a MagNA Lyser (Roche). Tissue homogenates were serially diluted and plated on Trypticase Soy Agar with 5% Sheep Blood (Eiken Chemical). Prior to transcardiac perfusion, blood was collected into heparin-containing tubes and plated on THY agar plates. For some experiments, mice were intranasally treated 24 h before pneumococcal infection with either the vehicle or 100 ng of recombinant PLY proteins in 10 µL of PBS, delivered as a 5-µL droplet per nostril.

### Real-time RT-PCR assay

Quantification of mRNA encoding cytokines and chemokines in the nasal lavage fluid and each brain segment was performed by real-time RT-PCR assay using primers listed in Table S1, as previously reported (38). Briefly, total RNA was isolated from the nasal lavage and brain tissues using RNeasy kit (QIAGEN) and RNeasy fibrous tissue minikit (QIAGEN), respectively. Synthesis of cDNA from total RNA was performed with a PrimeScript RT reagent Kit (TaKaRa). The possibility of DNA contamination was excluded by PCR analysis of non-RT samples. Primer sets for selected genes were designed using Primer Express software, version. 3.0 (Thermo Fisher Scientific). RT-PCR amplifications were performed using the SYBR Green method with an ABI StepOne Real-Time PCR system, v. 2.2 (Thermo Fisher Scientific). Relative expression amounts were calculated with the comparative threshold cycle (ΔΔ*C*_T_) method. The level of *gapdh* expression was used as an internal control.

### Histopathologic and immunohistochemistry examinations

Following perfusion fixation with 4% paraformaldehyde (PFA, Nacalai Tesque), brain tissue samples were obtained and fixed in 4% PFA at 4°C overnight. The samples were then embedded in paraffin, sectioned, and subjected to hematoxylin and eosin (HE) staining. Stained tissue sections were observed using an EVOS M5000 Imaging System (Thermo Fisher Scientific). The pathological analyses were performed by two clinicians blinded to the experimental grouping. Pathological features, including dense leukocytic infiltration in interstitial and alveolar spaces, hemorrhaging, vascular leakage, and edema formation, were graded as follows: 0 (none), 1 (mild), 2 (moderate), and 3 (severe). Disease score was defined as the sum of the individual pathological scores.

For immunohistochemistry (IHC) staining, the sections were incubated with a primary antibody against E-cadherin (rabbit mAb; Cell Signaling Technology) and a secondary antibody, Histofine Simple Stain MAX-PO (R) (Nichirei Biosciences). Peroxidase activity was visualized with the of a DAB kit (Vector). Imaging was performed using an EVOS M5000 Imaging System (Thermo Fisher Scientific).

Immunofluorescence staining was also performed, for which tissue sections were labeled with primary antibodies raised against serotype 19 capsule (rabbit pAb; Denka Seiken) and olfactory marker protein (goat pAb; FUJIFILM). After washing, the sections were incubated with Alexa Fluor 488-conjugated anti-rabbit IgG (Thermo Fisher Scientific) or Alexa Fluor 594-conjugated anti-goat IgG (Thermo Fisher Scientific), followed by staining with 4′,6-diamidino-2-phenylindole (DAPI) (Thermo Fisher Scientific). Imaging was performed using an FM10i confocal laser scanning microscope (Olympus).

### Statistical analysis

All statistical analyses were conducted using GraphPad Prism, v. 9.4.1 (GraphPad Software). Statistical analyses of the results were performed to determine significant differences using one-way analysis of variance (ANOVA), followed by Tukey’s multiple-comparison test. *P* values of less than 0.05 were considered to indicate statistical significance.

## Data Availability

The data supporting the findings of this study are available within the article and on request from the corresponding author.

## SUPPLEMENTAL MATERIAL

Fig. S1 msphere.00655-24-s0001.eps Pneumococcal dissemination into brain tissues.

Table S1 msphere.00655-24-s0002.docx Primer list.

ASM does not own the copyrights to Supplemental Material that may be linked to, or accessed through, an article. The authors have granted ASM a non-exclusive, world-wide license to publish the Supplemental Material files. Please contact the corresponding author directly for reuse.
