# Supplementary material for: Pneumolysin contributes to dysfunction of nasal epithelial barrier for promotion of pneumococcal dissemination into brain tissue
Source: mSphere. 2024 Sep 30;9(10):e00655-24. doi: 10.1128/msphere.00655-24 (PMC11520308; doi:10.1128/msphere.00655-24)
Supplement: Table S1 — Primer list. [file msphere.00655-24-s0002.docx]

**Takahara *et al.* Supplementary Table 1**

Table S1. Oligonucleotides used in this study.

Primer Sequence (5’-3’) Purpose

PLY_KO/F1 CTTTAGCCGTTTTCTTGGCTACG deletion of the *ply* gene

PLY_KO/R1 CTATTTAAATAACAGATTAAAAAAATTATAAGAGAGGAGAATGCTTGCGAC deletion of the *ply* gene

PLY_KO/F2 GTCGCAAGCATTCTCCTCTCTTATAATTTTTTTAATCTGTTATTTAAATAG deletion of the *ply* gene

PLY_KO/R2 CAGGAACTTATTAGGAGGTAGAAGATGAATACATACGAACAAATTAATAAAG deletion of the *ply* gene

PLY_KO/F3 CTTTATTAATTTGTTCGTATGTATTCATCTTCTACCTCCTAATAAGTTCCTG deletion of the *ply* gene

PLY_KO/R3 TTTTCTTTTACTATCAAATGTTTGAGC deletion of the *ply* gene

PLYKO_checkF TTTGGCTTTATCAATCGCTTTATCG confirmation of the *ply* deletion

PLYKO_checkR TAGAGGAAATGTCTCAATCCAGC confirmation of the *ply* deletion

PLY_compF1 CTATGGGCTTTCCTATTGATGAC complementation of the *ply* gene

PLY_compR1 GGATAGTAATTCATTCCTGGTTGGTCACCGCCGGTCGGGAATTTC complementation of the *ply* gene

PLY_compF2 GAAATTCCCGACCGGCGGTGACCAACCAGGAATGAATTACTATCC complementation of the *ply* gene

PLY_compR2 TATTTGCCATCTTCTACCTCCTAATTTATTTCCTCCCGTTAAATAATAGA complementation of the *ply* gene

PLY_compF3 TCTATTATTTAACGGGAGGAAATAAATTAGGAGGTAGAAGATGGCAAATA complementation of the *ply* gene

PLY_compR3 CTAGGCGACAAGCTGTAGGCCTAGTCATTTTCTACCTTATCTTCTAC complementation of the *ply* gene

PLY_compF4 GTAGAAGATAAGGTAGAAAATGACTAGGCCTACAGCTTGTCGCCTAG complementation of the *ply* gene

PLY_compR4 CCACCAGACCCACAAGGACTG complementation of the *ply* gene

PLY_compckF1 GAACCTCAAATTCCACAAAATACG confirmation of the *ply* complementation

PLY_compckR1 GTAGGAAACGAAGGGTTGTCCG confirmation of the *ply* complementation

PLY_compckF2 AAAAAGTGGTTTTTGAAAGCCATGC confirmation of the *ply* complementation

PLY_compckR2 CAAGCCTGGATGATCTGC confirmation of the *ply* complementation

rPLY-F CGCGGATCCATGGCAAATAAAGCAGTAAATGAC construction of rPLY

rPLY-R TTGCGGCCGCGTCATTTTCTACCTTATCTTCTACC construction of rPLY

rPLYW433F-FL1R CGTACGCCACCATTCAAAGGCAAGCCCGGTACA construction of rPLY_W433F

rPLYW433F-FL2F TGTACCGGGCTTGCCTTTGAATGGTGGCGTACG construction of rPLY_W433F

mCDH1-F ACCCCCTTACGACTCTCTGTTG real-time RT-PCR

mCDH1-R CAGGCTAGCGGCTTCAGAAC real-time RT-PCR

mSNAI1-F GAGCCCCAAGGCCGTAGA real-time RT-PCR

mSNAI1-R GAGCTTTTGCCACTGTCCTCAT real-time RT-PCR

mGli1-F CGCGCCTCTCCCACATACTA real-time RT-PCR

mGli1-R CGTCCCAACTGCTTCTTCATC real-time RT-PCR

mCXCL2-F GCGCCCAGACAGAAGTCATAG real-time RT-PCR

mCXCL2-R AGGGTCAAGGCAAACTTTTTGA real-time RT-PCR

mTNFA-F ACCCCCCCATGCTAAGTTCT real-time RT-PCR

mTNFA-R CCTGTGTCTATTTCCTTTTGATTTCTAA real-time RT-PCR

mCXCL1-F GCAGACCATGGCTGGGATT real-time RT-PCR

mCXCL1-R CCTGAGGGCAACACCTTCAA real-time RT-PCR

mCCL7-F GCTGCTTTCAGCATCCAAGTG real-time RT-PCR

mCCL7-R GCAGCATGTGGATGCATTG real-time RT-PCR

mIL1b-F TTGACGGACCCCAAAAGATG real-time RT-PCR

mIL1b-R TGGACAGCCCAGGTCAAAG real-time RT-PCR

mIL6-F CCACGGCCTTCCCTACTTC real-time RT-PCR

mIL6-R GAAGGAATGCCCATTAACAACAA real-time RT-PCR

IL10-F GATGCCCCAGGCAGAGAA real-time RT-PCR

IL10-R CACCCAGGGAATTCAAATGC real-time RT-PCR

mGAPDH-F CATGGCCTTCCGTGTTCCTA real-time RT-PCR

mGAPDH-R GCGGCACGTCAGATCCA real-time RT-PCR
